# The oculomotor signature of expected surprise

**DOI:** 10.1038/s41598-022-06403-4

**Published:** 2022-02-15

**Authors:** Dominika Drążyk, Marcus Missal

**Affiliations:** Institute of Neurosciences (IONS), Cognition and System (COSY), Université catholique de Louvain, 53 av Mounier, B1.53.04 COSY, 1200 Brussels, Belgium

**Keywords:** Neuroscience, Psychology

## Abstract

Expected surprise, defined as the anticipation of uncertainty associated with the occurrence of a future event, plays a major role in gaze shifting and spatial attention. In the present study, we analyzed its impact on oculomotor behavior. We hypothesized that the occurrence of anticipatory saccades could decrease with increasing expected surprise and that its influence on visually-guided responses could be different given the presence of sensory information and perhaps competitive attentional effects. This hypothesis was tested in humans using a saccadic reaction time task in which a cue indicated the future stimulus position. In the ‘no expected surprise’ condition, the visual target could appear only at one previously cued location. In other conditions, more likely future positions were cued with increasing expected surprise. Anticipation was more frequent and pupil size was larger in the ‘no expected surprise’ condition compared with all other conditions, probably due to increased arousal. The latency of visually-guided saccades increased linearly with the logarithm of surprise (following Hick’s law) but their maximum velocity repeated the arousal-related pattern. Therefore, expected surprise affects anticipatory and visually-guided responses differently. Moreover, these observations suggest a causal chain linking surprise, attention and saccades that could be disrupted in attentional or impulse control disorders.

## Introduction

Prediction of future events is an essential cognitive function that influences all levels of sensory and motor processing in the brain^1–3^. Prediction is based on prior information learned in a similar context. However, it is inevitably tainted with some spatial and/or temporal uncertainty that has to be explicitly estimated in the decision process^4^. Uncertainty comes in two different varieties: expected or unexpected. Expected uncertainty refers to the incorporation of the *unreliability* of prediction in a given environment into the decision process. Unexpected uncertainty refers to a change in the context itself altering all prior expectations^5^. It has been suggested that acetylcholine and noradrenaline could be involved in the processing of expected and unexpected uncertainty, respectively^5,6^. Both of these neurotransmitters also control pupil size^7^, which is commonly used as a behavioral measure of the subjective impact of uncertainty in sensory and decision processes^8–12^ (see review in^13^). Furthermore, uncertainty could also be qualified as ‘surprise’. Surprise is a polysemic term that can be interpreted in different ways and has been hypothesized to underlie essential functions like learning^14,15^ and emotions^16,17^. In the present study, we will use ‘surprise’ to quantify the unexpectedness of a particular event occurring from a random variable (‘stimulus-bound’ surprise^4^). Surprise will be computed using the definition from Shannon’s information theory as the negative logarithm of the probability that a target will appear at a given position and expressed in bits^18^. We will use the term ‘expected surprise’ to refer to the anticipation of the spatial uncertainty associated with a future visual target. The future position of the target will be provided by a cue creating a prior expectation but with a certain amount of associated spatial uncertainty. Although ‘expected surprise’ could be considered as an oxymoron (‘expecting the unexpected’), we suggest it has an operational validity. For instance, surprise is expected when you go home after work on the day of your birthday (prior knowledge) but you don’t know exactly what it will be (party, dinner out, etc.). Surprise also plays a major role in gaze shifting^19–21^ and could ‘capture’ oculomotor behavior^22^. Moreover, surprise attracts attention^23^ suggesting a causal chain between phenomena (surprise → attention → saccade^24^).

If surprise partly determines oculomotor behavior, it remains to be established how this phenomenon impacts different kinds of eye movements. Indeed, eye movements occur either in reaction to a stimulus in the environment and are sensory-guided or in anticipation of the appearance of an object of interest and are qualified as ‘anticipatory’. It has been shown that primates often anticipatorily gaze at the future expected position of a visual stimulus to collect information or anticipate action consequences^25^. In general, low uncertainty about sensory dimensions of a future stimulus favors anticipation in the oculomotor system (e.g. for smooth pursuit, see review in^26^). Anticipatory saccades are often observed in simple visual reaction time (RT) experiments, typically during the period between the warning and imperative stimuli. These saccades are considered as ‘uninstructed’ responses and are usually discarded from further analysis. Indeed, although sensory conditions favoring their occurrence could be determined (e.g. repetition of stimulus appearance at the same position) they occur spontaneously without strict experimental control and with large idiosyncratic variations^27^. Therefore they could be considered as errors. However, anticipatory saccades reflect the subject’s expectation and provide insights that cannot be obtained from stimulus-evoked responses only^28,29^. Indeed, uninstructed anticipatory responses are movements that are intentionally initiated under the influence of e.g. an expected reward or the expected position and timing of a future visual object. In contrast, visually-guided responses are instructed responses that are stimulus-bound. They are guided by the sensory evidence provided by the object of interest and a possible attentional competition between potential stimuli. Therefore, given the different information sources for these two behavioral responses, we hypothesized that surprise could have a different impact on anticipatory and visually-guided saccades. In the first case, the impact of surprise would be focused on internally-guided responses. In the second case, surprise could primarily affect sensorimotor processing and the spatial extent of attention. Surprise could also affect impulse control. Impulsivity is a multi-faceted concept including cognitive aspects and motor inhibition^30,31^. Motor inhibition is an essential cognitive function allowing to stop an impending or already started action and could be modulated by surprise. Indeed, it has been shown that cortico-spinal excitability (CSE) as measured using transcranial magnetic stimulation is globally reduced by a task-irrelevant unexpected (surprising) sound in a visually-guided Go/NoGo task^32^. A reduced CSE is a reliable indicator of increased motor inhibition that can be nonspecific and affect motor systems globally^33^. Eye movements are particularly sensitive to inhibitory control. Indeed, due to the weak inertia of the eye, the premotor system for saccades is kept under constant inhibition in order to avoid unintentional movements (see discussion in^34^). Any modulation of the intensity of this saccadic inhibitory ‘gate’ caused by surprise could change the probability to observe a saccade or its latency, even before the imperative stimulus.

In the present study, we investigated the influence of expected surprise on anticipatory, visually-guided saccades and pupil size. We hypothesized that the occurrence of anticipatory saccades could decrease with increasing expected surprise. Furthermore, the influence of expected surprise on visually-guided responses could be different given the presence of sensory information and perhaps competitive attentional effects. Pupil size could encode arousal related to surprise independently of other eye movements and indicate its subjective impact.

## Methods

### Subjects and ethics

Thirty-six subjects participated in the present study. One subject was excluded due to extensive noise in EEG and oculomotor recordings and one subject was excluded due to misunderstanding of the task. The final sample used in the analysis included 34 subjects (24 females; age = 24.50 ∓ 4.19 years). Participants were between 18 and 65 years old, did not suffer from neurological or psychiatric diseases, did not take drugs or psychoactive substances at least the day before the experiment and used corrected vision if needed. Each participant was informed about the aim of the study, signed an informed consent document regarding procedures and was informed about the possibility to withdraw from the experiment at any time without consequences. The study was conducted in accordance with the Declaration of Helsinki guidelines and approved by the local Ethics Committee of the Université catholique de Louvain under number B403201733677 (Belgium).

### Eye movements

Eye movements and pupil size were recorded binocularly at 500 Hz using an infrared eye tracking system (EyeLink1000, desktop mount, SR Research, Mississauga, Ontario) with an average spatial accuracy of 0.25° of visual angle and pupil size resolution of 0.2% of diameter. Experimental procedures were created using Experimental Builder (SR Research, Mississauga, Ontario) and displayed on a high resolution VPixx screen (1920 $\times$ 1080 pixels, VPixx Technologies, Canada) at 60 Hz. The eye-tracker camera was calibrated before each block of trials and after resting breaks.

### Experimental paradigm

**Figure 1:**
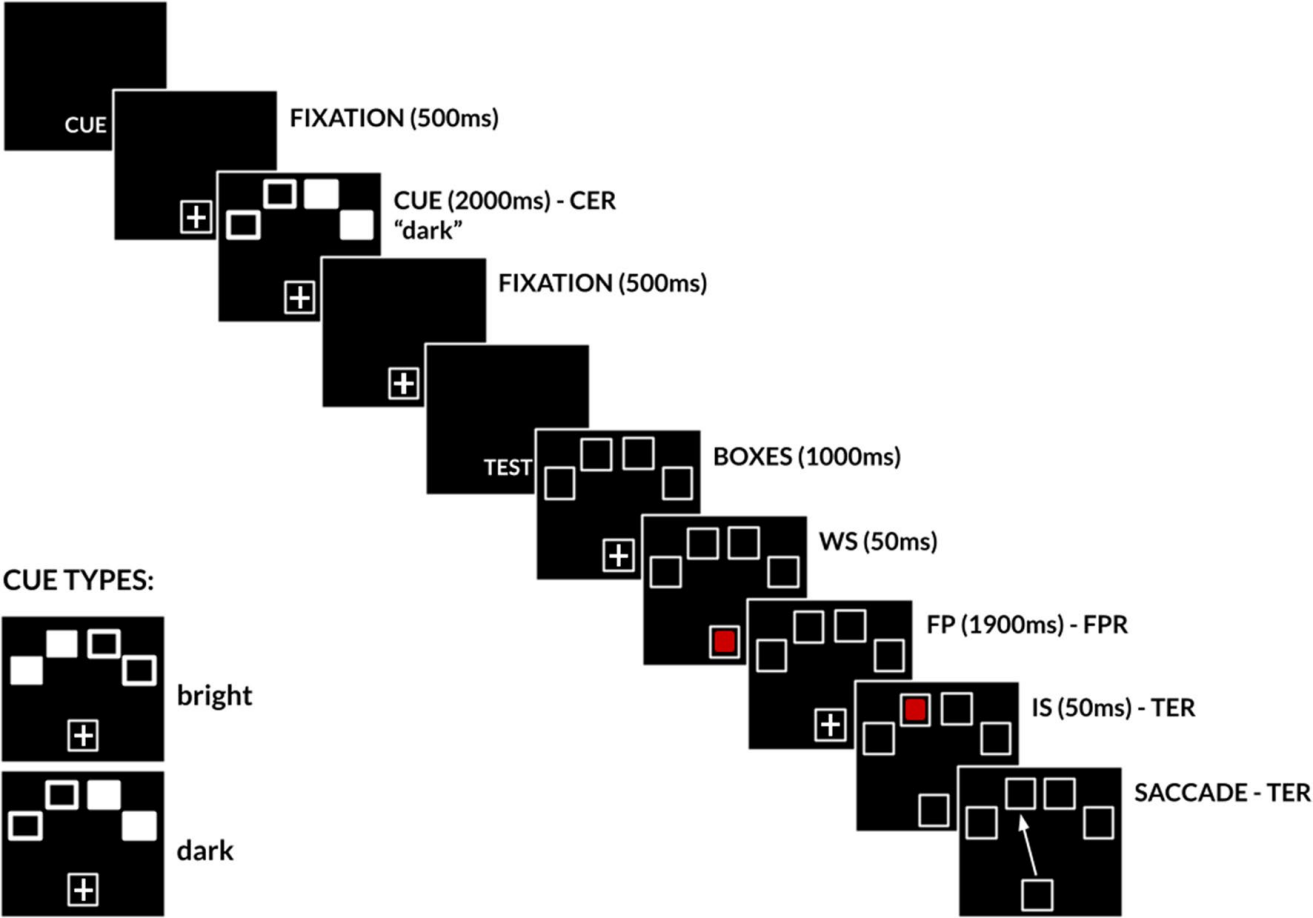
Schematic representation of the spatial surprise paradigm. During the cue interval, while maintaining gaze on the *fixation cross*, participants were presented with the spatial cue (cue boxes or CBs) on top of the screen. For half of the participants, CBs were represented as filled white squares (bright cue), for the other half open squares CBs were used (dark cue). CBs indicated the amount of expected surprise bounded to the future position of the target. Next, the warning stimulus (WS, first red square) was briefly presented in the *fixation box*. Extinction of the WS initiated a 1900 ms foreperiod (FP). Then the imperative stimulus (IS, second red square) was briefly presented in one of four *test boxes* (TBs). This event started the target-evoked response period (TER). Participants were asked to maintain gaze on the fixation cross during the FP and then make a visually guided saccade towards the IS as quickly as possible.

Each trial started with a cue period labeled by the word ’CUE’ displayed at the bottom of the screen (Fig. 1). Next, a white box (referred to as the *‘fixation box’*, 5.7 $\times$ 4.3° of visual angle) with a white fixation cross appeared at the bottom of the screen for 500 ms. After this initial fixation period a cue was presented. The cue consisted of four *cued boxes* (referred to as ‘CBs’; dark or bright; 5.7 $\times$ 4.3° of visual angle each) displayed above the fixation box for 2000 ms. The center of each cue box was at an approximate distance of 17° of visual angle from the center of the fixation cross. Participants were asked to maintain gaze fixation on the fixation box before, during and after the cue presentation.

Afterwards, the test period began with the word ’TEST’ presented and four empty *test boxes* (referred to as ‘TBs’, 5.7 $\times$ 4.3° of visual angle each) displayed on the top of the screen for 1000 ms at the same positions as CBs. The warning stimulus (WS, *red square*) was shortly displayed in the central box for 50 ms. After a constant delay of 1900 ms (*foreperiod* or FP) the imperative stimulus (IS, eccentric *red square*) was displayed in one of the TBs for 50 ms. Participants were instructed to maintain gaze on the fixation cross during the FP and then make a visually-guided saccade towards the IS as fast as possible. Each trial ended with an inter-trial-interval (ITI) of a randomized duration (2250 ∓ 250 ms). This paradigm has the advantage to clearly separate the expected surprise encoding period from the response preparation period. Indeed, the cue indicated the different levels of expected surprise about the future target position.

If the cue consisted of one marked box (n = 1), the probability of the target appearing in that box at the end of the foreperiod was P($\mathrm {TB}_1$) = 1 (no surprise). Note that for P($\mathrm {TB}_1$) = 1, the actual box being marked could be randomly assigned to one of four different possibilities on the screen. If 2 boxes were marked (n = 2), the target could later appear in either one of them with the same probability P($\mathrm {TB}_2$) = 0.5. The group of two cued boxes could occupy three different positions on the screen (left, as on Fig. 1, or middle or right). If three boxes were marked, the probability for each box to be occupied by the future target decreased to P($\mathrm {TB}_3$) = 0.33. The group of three boxes could appear either mostly on the left or on the right side of the screen. Finally, if 4 boxes were marked, the probability of each box to be occupied by the future target was P($\mathrm {TB}_4$) = 0.25. In summary:

$$\begin{aligned} P(TB_{n}) = \left\{ \begin{array}{rl} 1, &{} if \ n = 1 \\ 0.5, &{} if \ n = 2 \\ 0.33, &{} if \ n = 3 \\ 0.25, &{} if \ n = 4 \end{array} \right. \end{aligned}$$

where: $\mathrm {TB}_n$ = target appearing in one of n boxes; n = number of marked boxes, n $\mathrm {\in }$ {1, 2, 3, 4}.

The value of surprise (SU) bounded to each experimental condition ($\mathrm {TB}_n$) can be calculated using Shannon’s formula:

1 $$\begin{aligned} SU_n = - log_2P(TB_n) \end{aligned}$$

which is a negative base 2 logarithm of the probability of the target appearing on one of the marked boxes P($\mathrm {TB}_n$), giving a result in bits^18^. For example, in condition $\mathrm {TB}_1$, $\mathrm {SU}_{1}$ = 0 indicated no surprise concerning the outcome and participants were precisely informed about the box that will be occupied by the future IS. Oculomotor preparation was maximal. Surprise for each condition was: $\mathrm {SU}_1$ = 0, $\mathrm {SU}_{2}$ = 1, $\mathrm {SU}_{3}$ = 1.58 and $\mathrm {SU}_{4}$ = 2.

Two different versions of the experiment were tested. For half of the participants, CBs were represented as filled white squares (*bright* cue), for the other half open squares CBs were used (*dark* cue). The edges of bright and dark CBs were blurred to minimize the pop-up effect of a cue appearing at the top of the screen and facilitate maintaining the gaze on the fixation box (see Supplementary material A for examples). Finally, to make sure that participants were able to memorize cued locations while fixating, a simple spatial working memory task was conducted at the beginning of each experiment. First, participants had to maintain gaze on the fixation box and remember the location of marked CBs presented. Next, four TBs were presented and participants had to click with the mouse the boxes previously marked during the cue presentation. Each experiment started with the memory task (20 trials), followed by the saccade task (4 blocks, 40 trials each).

### Preprocessing

**Figure 2:**
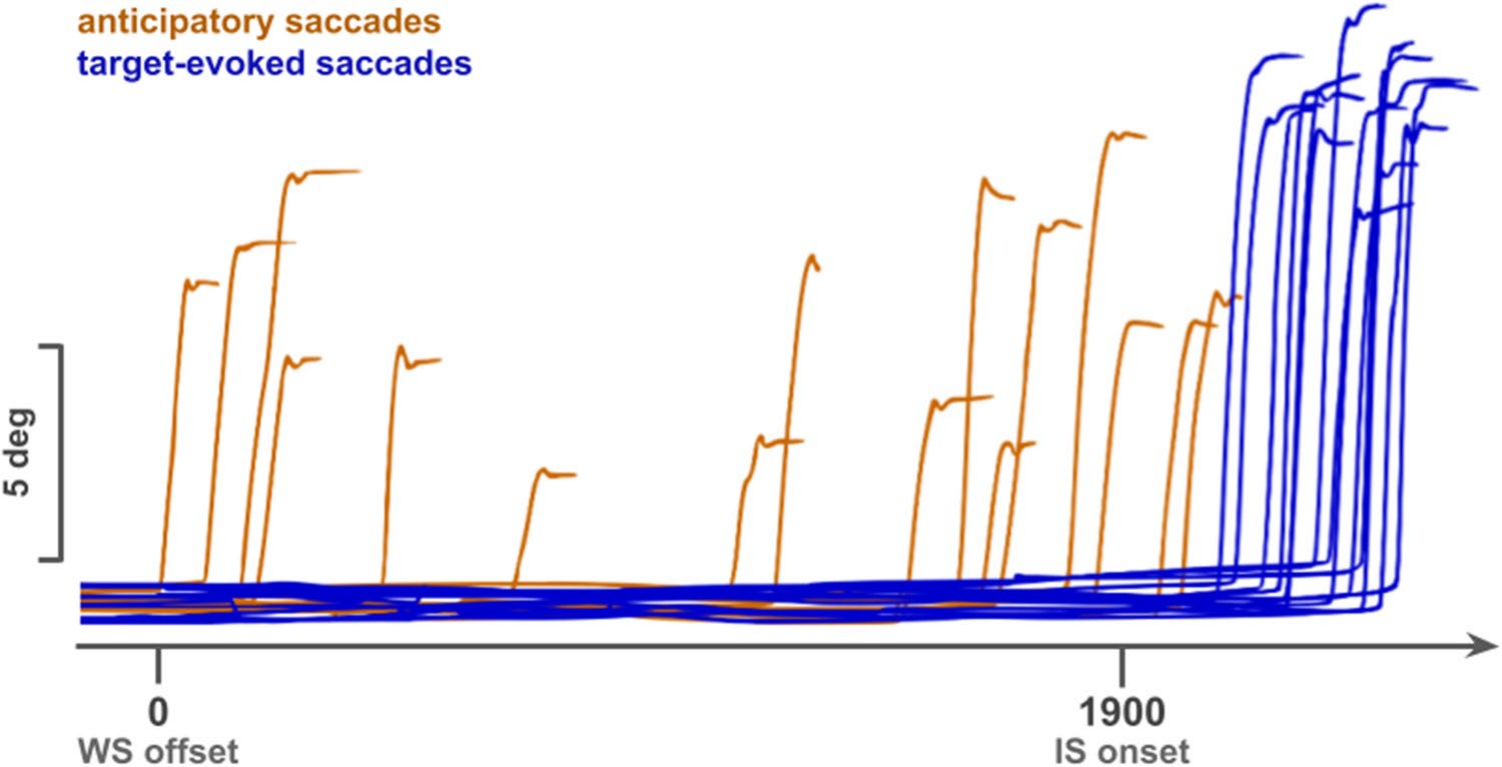
Example of anticipatory (orange traces) executed after the WS offset but not later than 100 ms after the IS onset, and visually guided (blue traces) saccades executed after 100 ms from the IS onset.

Poor quality oculomotor recordings (unreliable pupil tracking, frequent blinks) were excluded from the analysis using the DataViewer software (SR Research, Ontario, Canada). Only 4% of all recorded trials were excluded. After artifact rejection, saccades were detected in 96% of trials remaining with both a velocity (22°/s) and acceleration (3800°/$\mathrm {s}^{2}$) criteria. Saccades with a latency superior to or equal to 100 ms after appearance of the IS will be referred to as ‘visually-guided’ saccades (*blue traces* on Fig. 2). Saccades occurring after the extinction of the WS but less than 100 ms after the IS will be referred to as ‘anticipatory’ saccades (*orange traces* on Fig. 2; 5.50% of all trials remaining after artifact rejection). Visually-guided saccades with a latency > 1000 ms or executed after an anticipatory saccade or landing in the incorrect target box were not considered for further analysis (13% of all trials remaining after artefact rejection).

Pupil size during blinks was estimated by using a linear interpolation of pupil size from 70 ms before the starting point of the blink until 70 ms after its endpoint. Pupil diameter during the FP was then averaged in 50 ms bins and further processed in ‘R’ using the *PupillometryR* library (R Core Team, 2020, version 4.0.3^35^). Pupil traces were filtered using the median filter and normalized to the baseline period (100 ms before the data segment onset).

### Statistical analysis

Statistical analysis of multidimensional data is often associated with the multiple comparison problem (MCP). The cluster based permutation test (CBPT) is a popular data-driven approach that alleviates the MCP^36^. However, contrary to widespread use, this approach is not tailored to support claims about the precise location of the detected effects^37^. Therefore, each cluster-based permutation test result was further submitted to a hypothesis-driven method conducted on the clusters suggested by CBPT. In CBPT, t-values of the difference between two conditions were computed and thresholded (*p* < .05) to specify clusters of significant differences. Labels of trials were shuffled 10,000 times randomly and a t-test was performed on the shuffled data. The sum of t-values within the largest cluster was saved into a distribution of summed cluster t-values representing the null hypothesis. Next, to correct for multiple comparisons, non-permuted cluster t-values were summed and thresholded using the null-hypothesis distribution (*p* < .05). Pupil size changes during the FP were subjected to CBPT analysis in R (*permutes* library version 2.1.1^38^).

Within the hypothesis-driven approach, mean pupil size, number of anticipatory saccades, visually-guided saccadic maximum velocity and latencies were subjected to a statistical analysis using *lme4* library (version 1.1-23^39^). LMMs were fitted using the maximum likelihood method (Laplace Approximation^39^). A log-likelihood ratio test was conducted to compare models^40^. Each linear mixed effects model used in the manuscript can be explained by the general equation:

2 $$\begin{aligned} Y_{is} \sim \beta _{0} + \beta _{1}X_{is} + S_{0s} + \varepsilon _{is} \end{aligned}$$

where $\mathrm {Y}_{is}$ is the outcome for the *s*-th subject in the *i*-th trial, $\mathrm {X}_{is}$ is the fixed effect for the *s*-th subject in the *i*-th trial, $\beta _0$ is the intercept parameter, $\beta _1$is the fixed effect slope parameter, $\mathrm {S}_{0s}$ is the random intercept effect for subjects and $\varepsilon _{is}$ is the corresponding residual error. Continuous outcomes (visually-guided saccadic latencies, maximum velocity and pupil size) were fitted using Gaussian distributions. Count measures of anticipatory saccades were fitted using the Poisson distribution. Due to the large number of zeros in the measure, a zero-inflated (ZI) Poisson model was chosen. In this case, the excess zeros contribution included only an intercept term. Finally, accuracy of the memory task responses were fitted using a binomial distribution. Zero-inflated Poisson and the binomial distribution were used within the Generalized Linear Mixed Models (GLMM) framework. Outcomes and fixed effects specified for a given model are listed in the Results section. The significance level assumed in the present study was $\alpha$ = 0.01.

## Results

### Pupil size during the cue and foreperiod

**Figure 3:**
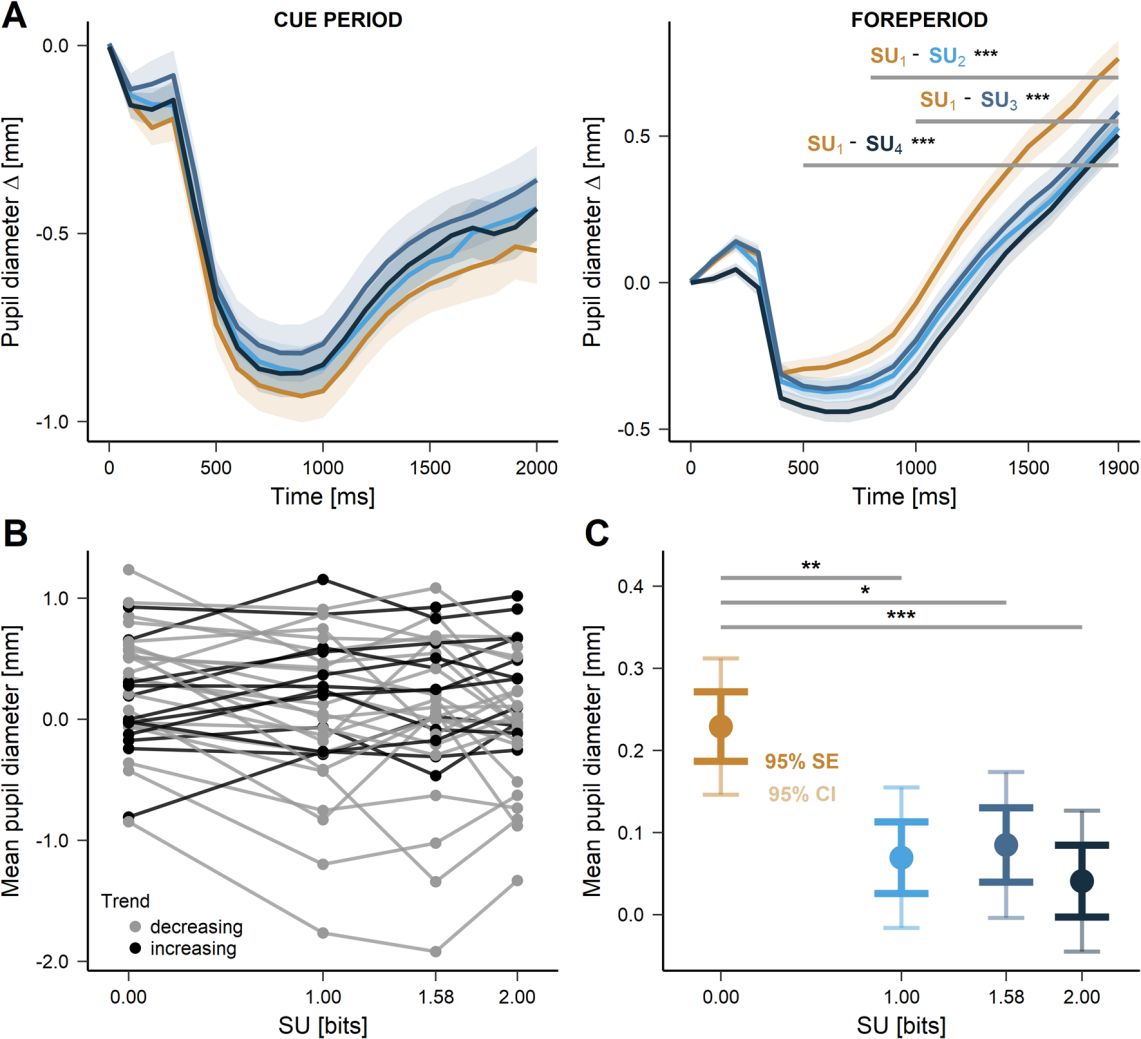
(**A**) Time course of pupil size during the cue period (left panel) and FP (right panel). Time ‘zero’ on the X-axis indicates the onset of the cue or the extinction of the warning stimulus. Colored lines indicate baseline corrected mean pupil size in the different SU conditions. Horizontal grey lines indicate clusters of differences between conditions, together with p-values obtained with the CBPT (*p $\mathrm {\leqslant }$ .05, **p $\mathrm {\leqslant }$ .01, ***p $\mathrm {\leqslant }$ .001). (**B**) Mean pupil size (dot) in each surprise condition in the 800–1900 ms FP interval for each subject. Black lines show individuals with an increasing trend, calculated as a difference between $\mathrm {SU}_{1}$ and $\mathrm {SU}_{4}$ conditions. (**C**) Mean pupil size (dot) for each SU context in the 800–1900 ms FP interval with standard errors and confidence intervals. Horizontal grey lines indicate LMM significance between conditions.

During the cue period, pupil size presented a biphasic time course starting with an initial transient response lasting approx. 500 ms followed by a sustained change lasting approximately 1500 ms (see Fig. 3A, left panel). In order to determine if the pupil response could encode expected surprise (abbreviated as ‘SU’) during the cue presentation period, a cluster-based permutation analysis was performed. None of the tested pairs of contrasts ($\mathrm {SU}_{1}$ - $\mathrm {SU}_{2}$, $\mathrm {SU}_1$ - $\mathrm {SU}_{3}$, $\mathrm {SU}_1$ - $\mathrm {SU}_{4}$) proved significantly different ($p _{mass}>$ .05). However, pupil size was modulated by the number of bright CBs (see Supplementary material A for figures).

Observation of pupil size as a function of time during the foreperiod (Fig. 3A, right panel) suggests here also that pupil dynamics up to 500 ms after the WS offset was probably related to luminance changes only. However, in contrast with the cue period, a significant modulation of pupil size by expected surprise was found using a cluster-based permutation analysis conducted for each pair of surprise conditions separately ($\mathrm {SU}_{1}$ and $\mathrm {SU}_{2}$: $t _{mass}$ = 86.32, $p _{mass}$ = .0001, interval 800–1900 ms; $\mathrm {SU}_{1}$ and $\mathrm {SU}_{3}$: $t _{mass}$ = 53.38, $p _{mass}$ = .0001, interval 1000–1900 ms; $\mathrm {SU}_{1}$ and $\mathrm {SU}_{4}$: $t _{mass}$ = 189.30, $p _{mass}$ = .0001, interval 500–1900 ms). The 800–1900 ms period was therefore prone to show an effect of SU on pupil size and subjected to further analysis.

**Table 1:** LMM analysis of the effect of expected surprise on the pupil size during the foreperiod.

Model	Effects	$\beta$	*SE*	*t*	*p*	*95% CI*	*df*	$\sigma ^2$	$\tau _{00}$	*LL*
A0	*Intercept*	0.12	0.09	1.30	0.200	[− 0.05, 0.31]	5184	2.23	0.26	− 9493
A1	*Intercept* ($SU_{1}$)	0.17	0.09	1.31	0.200	[− 0.05, 0.29]	5181	2.23	0.26	− 9487
	$SU_{1}$ - $SU_{2}$	0.16	0.06	2.71	**0.007**	[0.05, 0.28]				
	$SU_{1}$ - $SU_{3}$	0.15	0.06	2.50	**0.012**	[0.03, 0.25]				
	$SU_{1}$ - $SU_{4}$	0.19	0.06	3.22	**0.001**	[0.07, 0.30]				

$\beta$ fixed effect coefficient, *LL* Log-Likelihood, $\sigma ^2$ variance of level-1 residual errors, $\tau _{00}$ variance of level-2 residual errors.

Significant values are in bold.

A linear mixed effects analysis was performed according to the following model:

3 $$\begin{aligned} Model\;A1:\;{pupil\;size}_{is} \sim \beta _{0} + \beta _{1}\;SU_{is} + S_{0s} + \varepsilon _{is} \end{aligned}$$

with: $\mathrm {pupil\;size}_{is}$: mean pupil size for the *s*-th subject in the *i*-th trial within the 800–1900 ms interval; $\mathrm {SU}_{is}$ : value of surprise for the *s*-th subject in the *i*-th trial.

Contrasts were applied to the model in order to compare $\mathrm {SU}_{1}$ against each of the remaining conditions. Visual inspection of residual plots did not reveal any deviation from the model assumptions. Model A1 performed better, compared to an intercept-only based model A0 ($\mathrm {\chi }^2$(3) = 12.38, *p* = .006; see Table 1 for details). Within the 800-1900 ms interval of the foreperiod, pupil size was larger in the $\mathrm {SU}_{1}$ condition (mean ∓ std; 0.24 ∓ 0.10 mm) compared to all other conditions ($\mathrm {SU}_{2}$, 0.08 ∓ 0.10 mm, *p* = .007; $\mathrm {SU}_{3}$, 0.09 ∓ 0.10 mm, *p* = .012; $\mathrm {SU}_{4}$, 0.05 ∓ 0.10 mm, *p* = .001, see Fig. 3C), This trend was observed in most subjects of the present study (27/34, see Fig. 3B).

### Anticipatory oculomotor behavior and expected surprise

**Figure 4:**
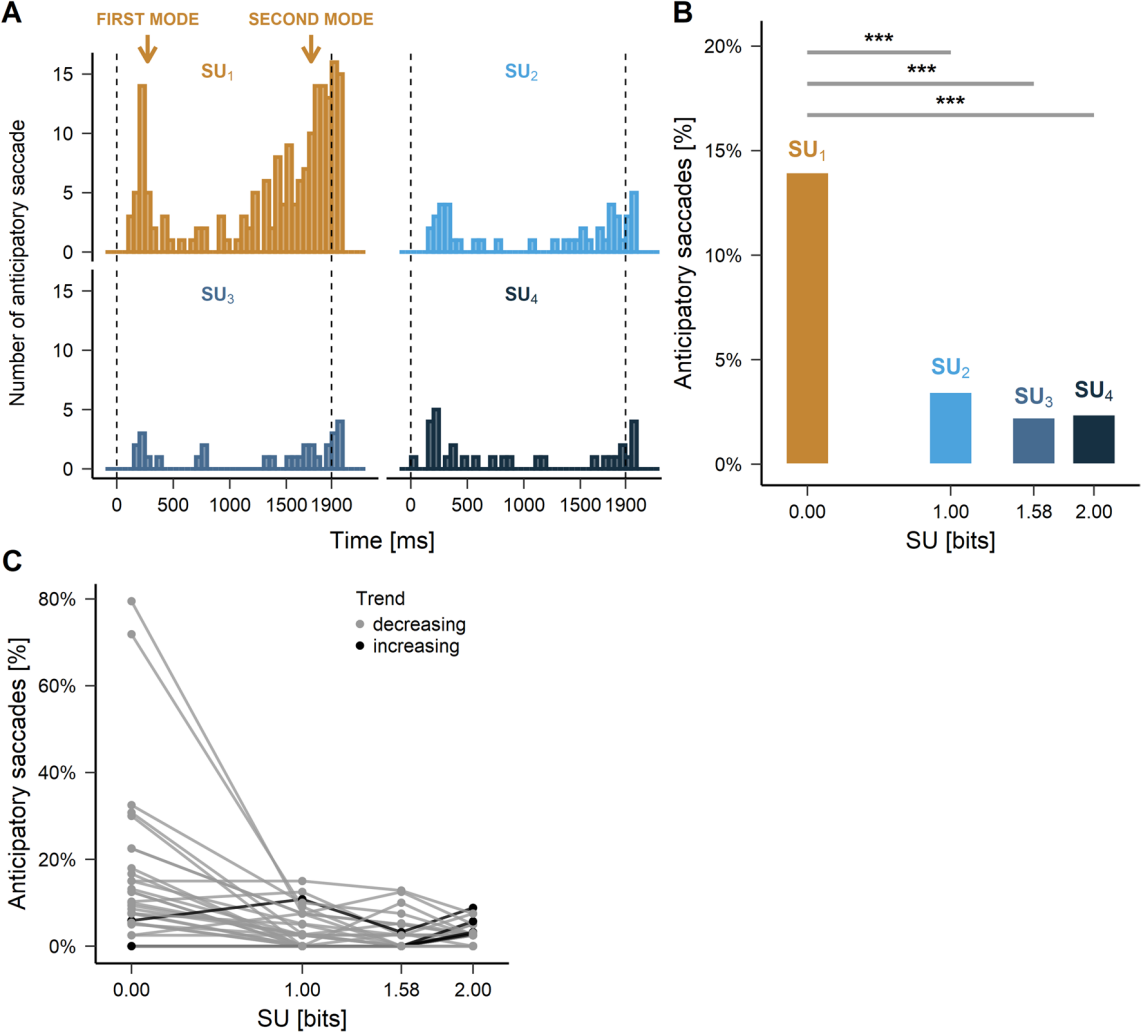
(**A**) Latency distributions of anticipatory saccades for the different SUs tested. Time ‘zero’ on the X-axis indicates FP onset. In the absence of surprise about the future target position, a bimodal distribution of latencies was clearly observed. (**B**) Bar plot of the percentage of anticipatory saccades during the FP. Horizontal grey lines indicate GLMM significance level between conditions. (**C**) Percentage of anticipatory saccades during the FP for each SU and each subject. Black lines show subject with an increasing number of anticipatory saccades with increasing SU (opposite trend). The trend was calculated as the difference between $\mathrm {SU}_{1}$ and $\mathrm {SU}_{4}$ conditions.

Saccades initiated during the foreperiod and up to 100 ms after the onset of the IS were defined as ‘anticipatory’ (see Fig. 2, *orange* traces). Figure 4A shows the latency distribution of anticipatory saccades for the different SU values tested. Visual inspection of saccadic latency distributions suggests the existence of two modes: an early one (0–500 ms) and a late one (> 1000 ms, see *arrows* on Fig. 4A). The first mode corresponds to a transient increase of the number of anticipatory saccades after the offset of the WS. The second mode shows a gradual increase of the probability of anticipatory saccade occurrence as time elapsed during the foreperiod. This anticipatory behavior was particularly salient in the $\mathrm {SU}_{1}$ condition. As SU increased, the number of anticipatory saccades decreased strongly and the bimodal pattern could only be conjectured. In order to quantitatively compare the number of anticipatory responses for different SUs, a generalized linear mixed-effects analysis was performed using model:

4 $$\begin{aligned} Model\;B1:\;{saccade\;count}_{s} \sim \beta _{0} + \beta _{1}\;SU_{s} + S_{0s} + \varepsilon _{s} \end{aligned}$$

with: $\mathrm {saccade\;count}_{s}$, number of anticipatory saccades for the *s*-th subject; $\mathrm {SU}_{s}$, surprise value for each subject. Contrasts were tested to allow the comparison of the number of anticipatory saccades in the $\mathrm {SU}_{1}$ condition against other SU values. Visual inspection of residual plots did not reveal any deviations from model assumptions or overdispersion. Model B1 performed better, compared to an intercept-only based model B0 ($\mathrm {\chi }^2$(3) = 130.85, *p* < .001, see Table 2 for details). During the FP, the occurrence of anticipatory saccades was 3.9 ∓ 0.7 times more likely in the $\mathrm {SU}_{1}$ compared with the $\mathrm {SU}_{2}$ condition (*p* < .001), 5.5 ∓ 1.3 times more likely in the $\mathrm {SU}_{1}$ compared with the $\mathrm {SU}_{3}$ condition (*p* < .001) and 5.8 ∓ 1.2 times more likely in the $\mathrm {SU}_{1}$ compared with the $\mathrm {SU}_{4}$ condition (*p* < .001, Fig. 4B, see Table 2 for details).

The amplitude of anticipatory saccades in the $\mathrm {SU}_1$ condition was 11.7° on average (*95% CI* [10.69, 12.67]) for first mode responses (n = 32) and 12.9° (*95% CI* [12.41, 13.36]) for second mode responses (n = 150). Saccadic amplitude was closer to target eccentricity (17°) in second mode responses (for the detailed information about saccade trajectory in different experimental conditions, see Supplementary material B). Due to unequal sample sizes, Dunnett’s test for multiple comparisons was performed on the saccadic amplitude in the first and second mode. The amplitude difference between modes (*t* = 2.25, *p* = .029) did not reach the significance level assumed in the present study ($\alpha$ = 0.01), probably due to the small sample size.

In summary, in response to no expected surprise about target location, the rate of anticipatory saccades during the FP was higher compared with all other conditions. Figure 4C shows that this trend was observed in most subjects.

**Table 2:** GLMM analysis of the effect of expected surprise on the anticipatory saccade count during the foreperiod.

Model	Effects	*IRR*	$\beta$	*SE*	*z*	*p*	*95% CI*	$\sigma ^2$	$\tau _{00}$	*LL*
B0	*Intercept*	2.22 $\mathrm {\pm }$ 0.43	0.80	0.19	4.14	<.**001**	[0.42, 1.18]	0.74	0.72	− 301
	*ZI part*	0.42 $\mathrm {\pm }$ 0.12	− 0.87	0.27	− 3.16	**.002**	[− 1.40, − 0.33]			
B1	*Intercept* ($SU_{1}$)	1.33 $\mathrm {\pm }$ 0.26	0.29	0.19	1.48	.140	[− 0.09, 0.67]	0.56	0.54	− 236
	$SU_{1}$ - $SU_{2}$	3.91 $\mathrm {\pm }$ 0.70	1.36	0.18	7.58	<**.001**	[1.01, 1.72]			
	$SU_{1}$ - $SU_{3}$	5.50 $\mathrm {\pm }$ 1.27	1.70	0.23	7.40	<**.001**	[1.25, 2.16]			
	$SU_{1}$ - $SU_{4}$	5.82 $\mathrm {\pm }$ 1.20	1.76	0.21	8.52	<**.001**	[1.36, 2.17]			
	*ZI part*	0.16 ${\pm }$ 0.09	-1.82	0.58	− 3.14	**.002**	[− 2.96, − 0.69]			

*IRR* incidence rate ratio $\mathrm {\pm }$ SE, $\beta$ fixed effect coefficient, *LL* Log-likelihood, $\sigma ^2$ variance of level-1 residual errors, $\tau _{00}$ variance of level-2 residual errors.

Significant values are in bold.

### Latency and maximum velocity of visually-guided saccades as a function of expected surprise

**Figure 5:**
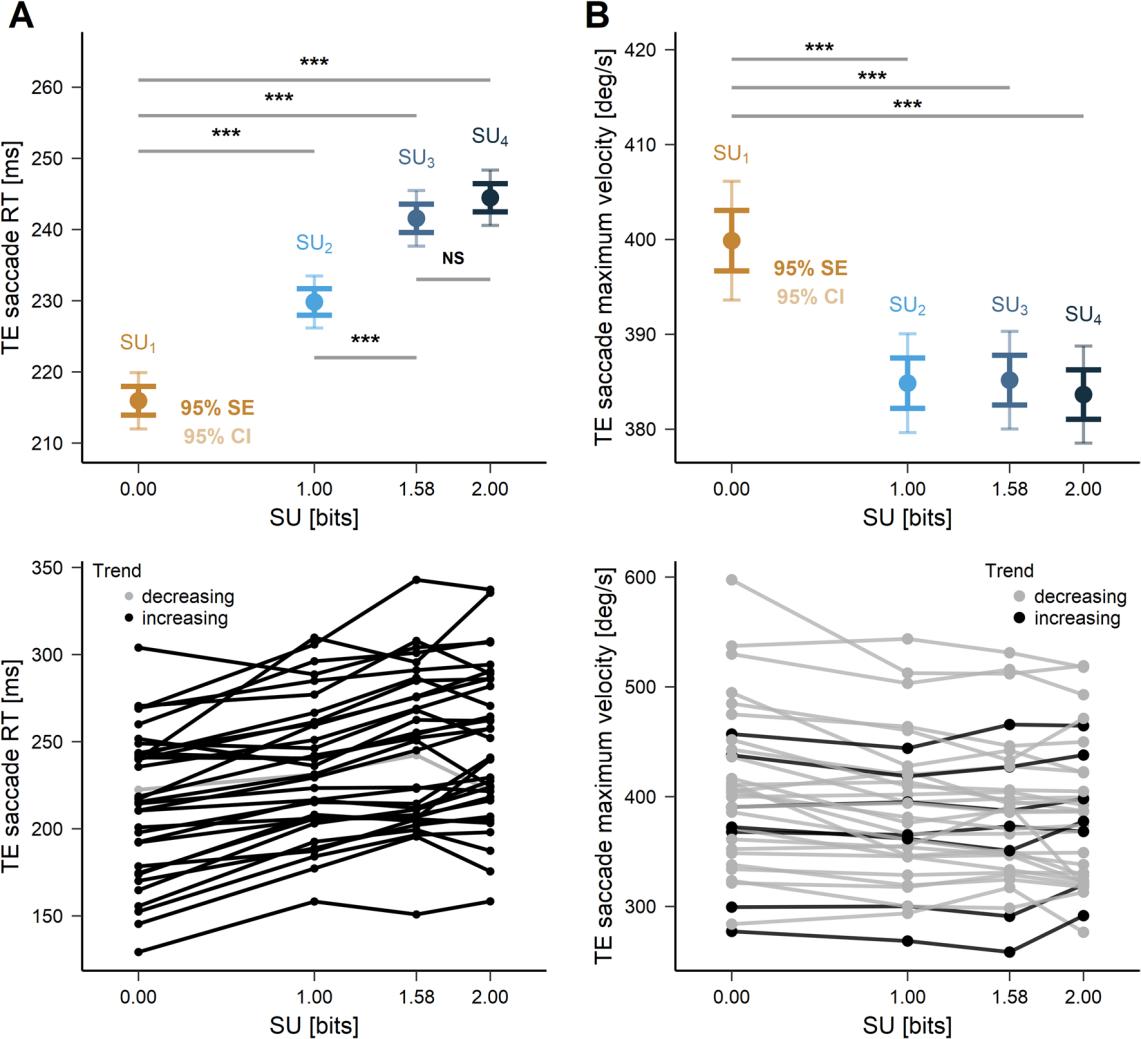
(**A**) Mean visually-guided RT (dots) for each SU context with standard error and confidence interval whiskers (upper panel) and divided by subject (bottom panel). Horizontal grey lines indicate LMM significance between conditions. Black lines show individuals with the decreasing trend. Different SU contexts are expressed in bits. (**B**) Mean visually-guided maximum velocity (dots) for each SU context with standard error and confidence interval whiskers (upper panel) and divided by subject (bottom panel). Horizontal grey lines indicate LMM significance between conditions. Black lines show individuals with the decreasing trend. Different SU contexts are expressed in bits. The trend was calculated as the difference between $\mathrm {SU}_{1}$ and $\mathrm {SU}_{4}$ conditions. Different SU contexts are expressed in bits.

In the Introduction section, we hypothesized that an internal representation of surprise could also alter the preparation of visually-guided saccades. Increasing surprise could alter movement latency and/or kinematics. In order to test these hypotheses, a linear mixed effects analysis was performed with the following model:

5 $$\begin{aligned} Model\;C1:\;{RT}_{is} \sim \beta _{0} + \beta _{1}\;SU_{is} + S_{0s} + \varepsilon _{is} \end{aligned}$$

where $\mathrm {RT}_{is}$ is the mean visually-guided saccadic reaction time (latency) for the *s*-th subject in the *i*-th trial and $\mathrm {SU}_{is}$ is the value of surprise for the *s*-th subject in the *i*-th trial. Visual inspection of residual plots did not reveal any deviation from model assumptions. Model C1 performed better, compared with an intercept-only based model C0 ($\mathrm {\chi }^2$(3) = 220.90, *p* < .001, see Table 3). Visually-guided saccades had a shorter latency in $\mathrm {SU}_{1}$ condition (215.8 ∓ 7.1 ms) compared with $\mathrm {SU}_{2}$ (233.5 ∓ 7.1 ms, *p* < .001), $\mathrm {SU}_{3}$ (244.8 ∓ 7.1 ms, *p* < .001) and $\mathrm {SU}_{4}$ conditions (247.8 ∓ 7.1 ms, *p* < .001). $\mathrm {SU}_{2}$ evoked shorter reaction times compared to $\mathrm {SU}_{3}$ (*p* < .001) but the comparison between $\mathrm {SU}_{3}$ and $\mathrm {SU}_{4}$ was not significant (*p* = .179, Fig. 5A).

**Table 3:** LMM analysis of the effect of expected surprise on the visually-guided saccade reaction time.

Model	Effects	$\beta$	*SE*	*t*	*p*	*95% CI*	*df*	$\sigma ^2$	$\tau _{00}$	*LL*
C0	*Intercept*	236.30	6.87	34.41	<**.001**	[223.62, 250.59]	4488	3087.03	1579.23	− 24486
C1	*Intercept* ($SU_{1}$)	235.45	6.95	33.85	<**.001**	[222.30, 250.15]	4485	2939.17	1621.30	− 24376
	$SU_{1}$ - $SU_{2}$	− 17.71	2.36	− 7.52	<**.001**	[− 22.15, − 12.54]				
	$SU_{1}$ - $SU_{3}$	− 29.02	2.34	− 12.38	<**.001**	[− 33.31, − 24.58]				
	$SU_{1}$ - $SU_{4}$	− 32.03	2.34	− 13.70	<**.001**	[− 37.05, − 26.85]				
	$SU_{2}$ - $SU_{3}$	− 11.31	2.26	− 5.01	<**.001**	[− 15.63, − 6.44]				
	$SU_{3}$ - $SU_{4}$	− 3.01	2.24	− 1.34	.180	[− 7.47, 1.63]				

$\beta$ fixed effect coefficient, *LL* Log-likelihood,$\sigma ^2$ variance of level-1 residual errors, $\tau _{00}$ variance of level-2 residual errors.

Significant values are in bold.

In order to test whether visually-guided saccadic maximum velocity followed the same trend, a linear mixed effects analysis was performed with the following model:

6 $$\begin{aligned} Model\;D1:\;{maximum\;velocity}_{is} \sim \beta _{0} + \beta _{1}\;SU_{is} + S_{0s} + \varepsilon _{is} \end{aligned}$$

with: $\mathrm {maximum\;velocity}_{is}$, mean visually-guided saccadic reaction time for the *s*-th subject in the *i*-th trial; $\mathrm {SU}_{is}$, surprise for the *s*-th subject in the *i*-th trial. Visual inspection of residual plots did not reveal any deviation from model assumptions. Model D1 performed better compared with an intercept-only base model D0 ($\mathrm {\chi }^2$(3) = 62.04, *p* < .001, see Table 4). Maximum velocity was larger in $\mathrm {SU}_{1}$ condition (402.8 ∓ 11.2°/s) compared with $\mathrm {SU}_{2}$ (385.6 ∓ 11.2°/s, *p* < .001), $\mathrm {SU}_{3}$ (384.5 ∓ 11.2°/s, *p* < .001) and $\mathrm {SU}_{4}$ conditions (382.2 ∓ 11.2°/s, *p* < .001, Fig. 5B).

**Table 4:** LMM analysis of the effect of expected surprise on the maximum velocity of the visually-guided saccad.

Model	Effects	$\beta$	*SE*	*t*	*p*	*95% CI*	*df*	$\sigma ^2$	$\tau _{00}$	*LL*
D0	*Intercept*	388.15	11.06	35.09	<**.001**	[368.313, 410.071]	4507	4527.24	4123.87	− 25459
D1	*Intercept* ($SU_{1}$)	388.76	11.09	35.04	<**.001**	[366.14, 408.91]	4504	4467.71	4148.97	− 25423
	$SU_{1}$ - $SU_{2}$	17.27	2.90	5.95	<**.001**	[11.73, 23.20]				
	$SU_{1}$ - $SU_{3}$	18.38	2.89	6.35	<**.001**	[12.45, 24.58]				
	$SU_{1}$ - $SU_{4}$	20.68	2.89	7.17	<.**001**	[14.55, 25.91]				

$\beta$ fixed effect coefficient, *LL* log-likelihood, $\sigma ^2$ variance of level-1 residual errors, $\tau _{00}$ variance of level-2 residual errors.

Significant values are in bold.

**Figure 6:**
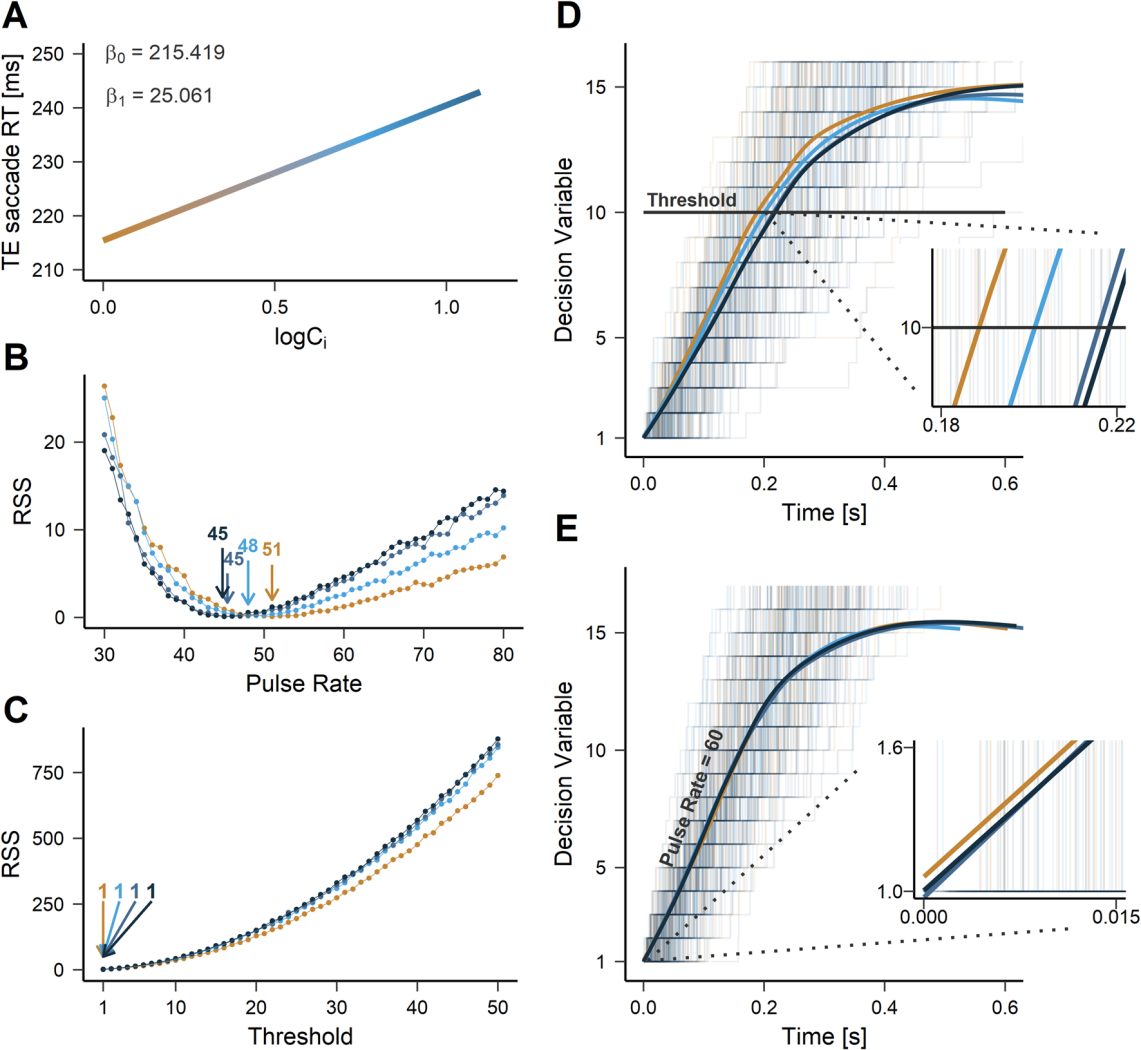
(**A**) Fit of the Hick’s model to saccadic reaction time. Predicted visually-guided RT plotted as a function of log(number of choices). (**B**) Residual sum of squares (RSS) of the comparison between empirical data and $\mathrm {R}_V$/$\mathrm {T}_C$ predictions. The plot was U-shaped with a variable minimum between conditions. (**C**) RSS of the comparison between empirical data and $\mathrm {R}_C$/$\mathrm {T}_V$ predictions. The RSS plot was exponentially shaped with a fixed arbitrary minimum identical in all conditions. (**D**) $\mathrm {R}_V$/$\mathrm {T}_C$ model. Fifty simulations trials (light traces) per condition are displayed with the mean path for every condition (dark lines). The horizontal line indicates the constant threshold. (**E**) $\mathrm {R}_C$/$\mathrm {T}_V$ model. See text for details.

The observation that visually-guided saccadic latencies logarithmically increased with expected surprise suggest a particular instantiation of Hick’s law^41^:

7 $$\begin{aligned} Model\;HL:\;{RT}_{i} \sim \beta _{0} + \beta _{1}\;log_{2}(C_i) \end{aligned}$$

with: $\mathrm {RT}_{i}$ average saccadic latency in the *i*-th trial; $\mathrm {\beta } _0$, intercept of the model; $\mathrm {\beta }_1$ slope; $\mathrm {C}_i$, number of choice alternatives for the *i*-th trial. Visual inspection of residual plots did not reveal any deviation from model assumptions. A significant regression was found between SUs and RT (*F*[1,134] = 13.47, *p* < .001) with an $\mathrm {R}^2$ of 0.085. Reaction time of visually-guided saccades increased by 25.1 ms for each unit of the logarithm of expected surprise (Fig. 6A).

Further, we tested whether visually-guided saccadic latencies could be predicted by a Poisson counting model^42–44^ (see implementation in^45^). This model is using a random walk to represent an hypothetical decision signal building up to a threshold and leading to a saccade. Each sample could be considered as a unit of evidence approaching the decision boundary. Samples were drawn from an exponential distribution and counted using a Poisson counting model. The accumulation average rate determines how quickly the decision threshold could be crossed. Two variants of the model were tested: in the ‘$\mathrm {R}_V$/$\mathrm {T}_C$’ model, the rate of evidence accumulation R was variable and could encode the different SUs tested. In this case, an arbitrary fixed threshold T was selected (see Fig. 6D); in the ‘$\mathrm {R}_C$/$\mathrm {T}_V$’ model, the rate of evidence accumulation was constant but the decision threshold could vary to encode SU (see Fig. 6E).

The $\mathrm {R}_V$/$\mathrm {T}_C$ model was fitted by simulating latencies of each SU condition separately with a threshold fixed at 10 units of accumulated evidence and an accumulation rate varying between 30 and 80 evidence units/s. Each fit was then compared to the average saccadic latency corresponding to each condition. The residual sum of squares (RSS) between simulated and observed data was then calculated and compared between fits. Figure 6B shows that the RSS plotted as a function of accumulation rate was U-shaped and reached a minimum at 51, 48, 45 and 45 units/s for the $\mathrm {SU}_{1}$, $\mathrm {SU}_{2}$, $\mathrm {SU}_{3}$ and $\mathrm {SU}_{4}$ condition, respectively. The rate of evidence accumulation was therefore different for each tested SUs. For the $\mathrm {R}_C$/$\mathrm {T}_V$ model, average saccadic latencies were simulated with a constant accumulation rate of 60 units/s and a range of possible thresholds values from 1 to 50 units. Figure 6C shows that the RSS for each fit plotted against the corresponding threshold value created an exponential function. This model produced larger RSS values than the $\mathrm {R}_V$/$\mathrm {T}_C$ model and provided an unrealistic fit of the data given that it did not properly differentiate the influence of SUs on saccadic latencies (except for the $\mathrm {SU}_{1}$ condition).

### Memory task

It could be hypothesized that observed effects are due to the number of items that subjects had to keep in declarative memory. More specifically, in multiple-choice reaction time tasks, the retrieval of information from declarative memory could explain HL, as suggested by the model of Schneider and Anderson^46^. Therefore, the effect of surprise and memory load could be confounded, even if there was only one response choice in the paradigm tested here. In order to test this possibility, a simple explicit spatial working memory variant of the main task was designed. First, participants were presented with the cue as in the main experiment and explicitly asked to remember the marked locations while looking at the fixation cross. Next, the array of four TBs appeared on the screen. The task consisted in recalling the previously presented CBs and selecting them with a mouse click while still fixating. For example, when the cue included 3 CBs (number of memory items = 3, abbreviated as ‘$\mathrm {MI}_{3}$’), participants had to click on the same 3 TBs during the response period. The memory experiment included 20 trials for each subject (5 per MI condition). Average accuracy was 97.6% ($\mathrm {MI}_{1} -$ 98.2%; $\mathrm {MI}_{2} -$ 96.5%; $\mathrm {MI}_{3} -$ 92.9%; $\mathrm {MI}_{4} -$ 98.2%). To quantitatively test the influence of the number of memory items on selection accuracy, a generalized linear mixed model analysis was performed with a binomial distribution:

8 $$\begin{aligned} Model\;E1:\;{accuracy}_{is} \sim \beta _{0} + \beta _{1}\;MI_{is} + S_{0s} + \varepsilon _{is} \end{aligned}$$

where $\mathrm {accuracy}_{is}$ is the binary performance indicator (1 - correct, 0 - incorrect answer) for the *s*-th subject in the *i*-th trial and $\mathrm {MI}_{is}$ is the number of locations to remember for the *s*-th subject in the *i*-th trial. Visual inspection of residual plots did not reveal any deviation from model assumptions. However, model E1 did not perform better than the corresponding intercept-based model E0 ($\mathrm {\chi }^2$(1) = 0.86, *p* = .354; see Table 5). Statistically, there was no influence of CB number on memory accuracy.

In conclusion, spatial working memory accuracy was similar across surprise conditions. Therefore, the logarithmic increase of visually-guided saccadic latency with surprise could not be simply attributed to an increasing memory load or task difficulty.

**Table 5:** GLMM analysis, influence of the number of memory items on the selection accuracy.

Model	Effects	$\beta$	*SE*	*z*	*p*	*95% CI*	$\sigma ^2$	$\tau _{00}$	*LL*
E0	*Intercept*	4.57	0.70	6.56	<**.001**	[3.20, 5.93]	3.29	2.79	− 87
E1	*Intercept*	5.08	0.91	5.58	<**.001**	[3.29, 6.86]	3.29	2.82	− 86
	*MI*	− 0.19	0.21	− 0.92	.360	[− 0.60, 0.22]			

$\beta$ fixed effect coefficient, *LL* log-likelihood, $\sigma ^2$ variance of level-1 residual errors,$\tau _{00}$ variance of level-2 residual errors.

Significant values are in bold.

## Discussion

In the present study, a simple oculomotor task was developed where subjects were informed about expected surprise with a spatial cue. When there was no expected surprise ($\mathrm {SU}_{1}$ condition, 0 bits), anticipatory saccades were more often observed and pupil size was larger. However, these responses did not co-vary with surprise for values > 0 bits. In contrast, the latency of visually-guided saccades regularly increased in a logarithmic manner with increasing expected surprise. This trend could be interpreted as a particular instantiation of Hick’s law (abbreviated as ‘HL’^41^). Unexpectedly, the maximum velocity of the same visually-guided saccades did not follow the same trend and was larger when surprise was 0 bits.

We suggest that the ‘no expected surprise’ condition ($\mathrm {SU}_{1}$ condition, 0 bits) favored anticipation due to an increased arousal level in the absence of uncertainty. In contrast, the abrupt decrease of anticipatory responses observed for higher expected surprise values ($\mathrm {SU}_{2}$, $\mathrm {SU}_{3}$, $\mathrm {SU}_{4}$) could be a consequence of a “Do not bet on the unknown” strategy reducing exploration^47^. Indeed, not knowing precisely what could happen could activate behavioral inhibition^48,49^. In the present study, the $\mathrm {SU}_{2}$, $\mathrm {SU}_{3}$ and $\mathrm {SU}_{4}$ conditions were associated with positional unexpectedness whereas in the $\mathrm {SU}_{1}$ condition the final target position was fully determined. Therefore, global motor inhibition could be stronger for SU > 0, resulting in a reduced probability of anticipatory responses. Global motor inhibition, including the oculomotor system, could occur automatically in conditions of uncertainty. Surprise in response to uncertainty and inhibition could have been tightly coupled during evolution^32^.

Interestingly, the latency distribution of anticipatory saccades during the foreperiod was bimodal with both an early and a late frequency increase (Fig. 4A; see $\mathrm {SU}_{1}$ condition). We suggest that the first mode of this distribution was constituted of premature impulsive saccades that were evoked by the extinction of the WS. Accordingly, their amplitude tended to be more variable. The second mode of the distribution was constituted of truly intentional anticipatory responses with the proper amplitude to reach one of the eccentric boxes. Importantly, the rate of anticipatory saccades in the second mode increased as time elapsed during the FP suggesting increasing temporal expectation of the IS, perhaps due to an increasing hazard rate of target onset^50^. Responses in both modes were reduced for SU > 0 bits, suggesting a strong top-down inhibitory process.

Pupil size was also larger in the $\mathrm {SU}_{1}$ condition. This finding is in agreement with previously published observations about pupil size and surprise^8–12^ (see^13^). However, in the present study, there was no gradual increase of pupil size with SU, suggesting that the $\mathrm {SU}_{1}$ condition specifically increased arousal. The higher impact of $\mathrm {SU}_{1}$ could also be due to the fact that the no surprise condition was globally less frequent than surprising conditions considered together ($\mathrm {SU}_{1}$, P = 0.25; { $\mathrm {SU}_{2}$, $\mathrm {SU}_{3}$, $\mathrm {SU}_{4}$}, P = 0.75). Therefore, arousal could be inversely related to the probability density of the low uncertainty event. In summary, we suggest that the SU_1_ condition was behaviorally more salient and increased arousal. As a consequence, pupil size increased and more anticipatory responses were evoked.

In contrast with anticipatory saccades and pupil size, the relationship between expected surprise and visually-guided saccade latency could be described using Hick’s Law^41,51^ (see review in^52^). In Hick’s original experimental paradigm, human subjects had to associate a key on a keyboard with the spatial position of a visual target. This task requires the association between a set of stimuli and a set of responses that is characteristic of cognitive operations in general. Hick’s law states that increasing the number of response alternatives (uncertainty) will cause a logarithmic increase of reaction time. If a base 2 logarithm is used, HL provides a value in bits that can be interpreted as a measure of information transfer in Shannon’s sense. Hick’s law has been repeatedly observed in different sensorimotor modalities. So far, evidence suggests that visually-guided saccadic eye movements obey HL if there is a choice of a cued target amongst several alternatives^53^ but it is not observed if saccades are simply visually-guided^54^. In contrast, anti-saccades latency obeys HL probably because the stimulus-response mapping in this task requires the cognitive effort of suppressing a saccade to the visual target and aiming at the opposite position^54^. We found that HL robustly fitted the relationship between average latency of visually-guided saccades and expected surprise. This observation was unexpected given that there was only *one* choice for the final response (the single visual target). Simply cuing expected surprise could have induced HL in saccadic latencies. Therefore, we suggest that HL could be observed even if there is no complex stimulus-response mapping but just expected surprise in memory. Schneider and Anderson^46^ suggested that HL could arise due to interferences during the retrieval of stimulus-responses associations from declarative memory in multiple-choice decision tasks. In the present study, the number of significant items during the cue presentation period necessarily increased with expected surprise potentially creating mnesic interferences. However, in a control task with explicit retrieval from memory, no significant effect of set size on the accuracy of memory retrieval was found. Therefore, we suggest that in the present study interferences during memory retrieval did not play a significant role but expected surprise per se could explain observed effects.

An alternative interpretation would be to suggest that attentional competition between potential saccadic goals could lead to HL in visually-guided saccades. Spatial theories of attention suggest that it could be oriented towards the saccade goal before movement onset. If several potential target positions are present simultaneously then attention could be allocated in parallel to these different saccade goals (see^55,56^). Therefore, in experiments reported here, when the imperative target appeared but before movement initiation, the *non-selected* attentional goals could compete for attentional resources and should be suppressed. This process could take more time if there are more potential goals with increasing expected surprise, leading to an increasing saccadic latency and HL. Similarly, the absence of attentional competition between goals in the $\mathrm {SU}_{1}$ condition could explain why anticipatory saccades were more frequent and visually-guided latencies shorter. This observation supports the hypothesis of a causal chain involving surprise → attention → saccade. In contrast with latency, saccadic maximum velocity as a function of SU could not be predicted with HL. In the oculomotor domain, the maximum velocity of saccadic eye movements is considered as a reliable indicator of arousal (review in^57,58^). In the present study, peak eye velocity was maximum in the no-surprise condition and then abruptly decreased for higher SUs. This result confirms the hypothesis of a central role of arousal ($\mathrm {SU}_{1}$ condition) together with another process that manifests itself on visually-guided saccadic latencies for SU > 0 bits ($\mathrm {SU}_{2}$, $\mathrm {SU}_{3}$, $\mathrm {SU}_{4}$). Recently, a relationship between pupil size and maximum eye velocity was found^59,60^. A subcortical mechanism involving the superior colliculus (‘SC’) could explain this relationship given its role in determining both saccade metrics and pupil size (reviews in^60–62^).

One standard way to model decision processes in psychology is a random walk to a threshold. This approach has encountered enormous success in modeling multiple-choice tasks when there is progressive accumulation of sensory evidence^43,63^. In the present study, there was no sensory evidence accumulation during the FP and only one choice. Therefore, we used a single threshold Poisson counting model that suggests that expected surprise could be encoded by the rate of rise of a decision signal ($\mathrm {R}_V$/$\mathrm {T}_C$ model). This simple model predicted observed saccadic latencies with a variance accounted for ($\mathrm {R}^{2}$) of 0.09 that was higher than if a fixed threshold with a variable rate was postulated ($\mathrm {R}_C$/$\mathrm {T}_V$ model). The observation that the $\mathrm {R}_V$/$\mathrm {T}_C$ model yields HL is not in agreement with previous modeling studies of HL where a criterion adjustment was found to be more likely^53,64^. Here also, this difference could be explained by the fact that there was no choice amongst several alternatives in the experimental design used here. In a context of cued expected surprise, HL could be observed even in a simple reaction time experiment with a single stimulus-response alternative. We dissociated surprise from complex stimulus-response mappings and nevertheless found HL (see^65^). In summary, the present study suggests two different processes involved in saccade preparation in a context of expected surprise: one related to arousal not obeying HL (pupil size, maximum velocity and anticipatory saccade occurrence) and the other one related to cognitive information processing obeying HL. We suggest that the arousal-related process could be partly determined by SC activity whereas the second process could explicitly encode expected surprise and be cortically controlled. Therefore, the proposed experimental paradigm might be particularly useful in the evaluation of neuropsychiatric disorders of impulse control and attention.

## Supplementary Information

Supplementary Information.

## Data Availability

The datasets generated during and/or analyzed during the current study are available from the corresponding author on reasonable request.
