# Supplementary material for: The oculomotor signature of expected surprise
Source: Sci Rep. 2022 Feb 15;12:2543. doi: 10.1038/s41598-022-06403-4 (PMC8847614; doi:10.1038/s41598-022-06403-4)
Supplement: Supplementary file 1 — Supplementary Information. [file 41598_2022_6403_MOESM1_ESM.pdf]

Supplementary material: The oculomotor signature of expected surprise

Dominika Drążyk[1,+] **and Marcus Missal**[1,*,+]

[1]Institute of Neurosciences (IONS), Cognition and System (COSY), Université catholique de Louvain, 53 av Mounier, B1.53.04 COSY, 1200 Brussels, Belgium
[*]marcus.missal@uclouvain.be
[+]these authors contributed equally to this work

A. Pupillary response during cue period depends on the visual intensity of the cue.

Two different versions of the task were introduced in order to account for possible differences in pupillary responses during the cue interval that could be caused by the number of visually intense bright cue boxes. Therefore, for half of the participants cue boxes encoding possible future target locations were bright and for the other half they were dark (see Fig. S1 for the examples). This procedure allows to dissociate the putative influence of expected surprise from cue brightness during the cue period.

During the cue interval (Fig. S2A), as expected pupil size time course was different in the dark (left panel) and bright (right panel) conditions. In the dark cue version (left panel), the pupil dilated more in response to the maximal surprise SU_4 condition and less in the SU_1 condition (when the image contained three bright CBs). In the bright cue version (right panel), the pupil dilated more in response to SU_1 (one bright cue box) and less in SU_4 when all the cue boxes were bright. A cluster-based permutation analysis was performed on the cue interval for every pair of SU conditions in the two task versions separately. The difference between surprise conditions was detected for all three pairs of SUs in the dark cue version (SU_1 and SU_2 pair, t_{mass} = 29.57, p_{mass} = .0002, interval 500-1000 ms; SU_1 and SU_3 pair, t_{mass} = 132.24, p_{mass} = .0002, interval 400-1700 ms; SU_1 and SU_4 pair, t_{mass} = 323.60, p_{mass} = .0002, interval 400-2000 ms). For the bright cue version, only the pair of extreme conditions (SU_1 and SU_4) presented a significantly different interval of pupil size (t_{mass} = 210.51, p_{mass} = .0002, interval 400-1600 ms). We therefore conclude that the time course of pupil response during the cue interval was strongly driven by the brightness of the presented cue. This effect did not transfer to the foreperiod interval. Indeed, the time course of pupil size during the foreperiod (Fig. S2B) shows that the influence of SU was not affected by cue brightness during the preceding cue period.

The brightness of the cue neither influenced the amplitude nor the direction of foreperiod saccades. Figure S3A shows the position of the eye at the beginning and end of anticipatory saccades for the two task versions. Visual inspection shows no difference between the trajectories of foreperiod saccades executed in the dark or bright versions of the experiment, neither for the first nor for the second mode. In addition, the brightness of the cue did not alter saccade trajectory in the different SU conditions (Fig. S3B).

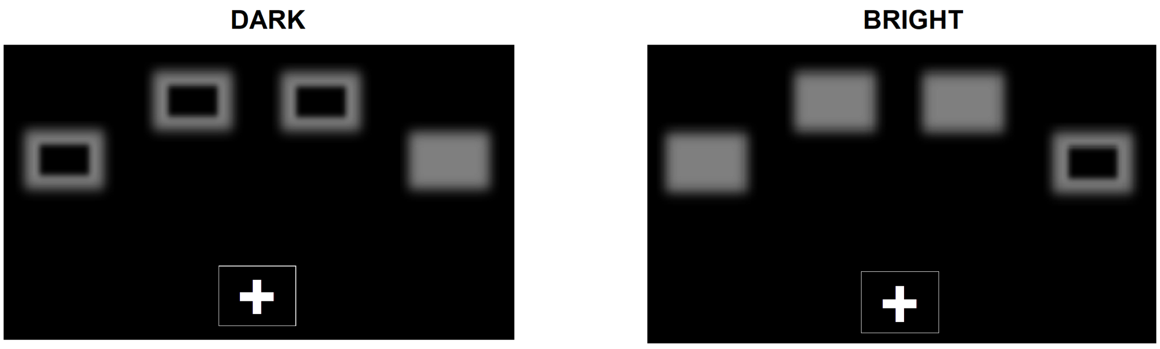

Figure S1. Examples of cues in the *dark* and *bright* versions of the task (SU_3 condition).

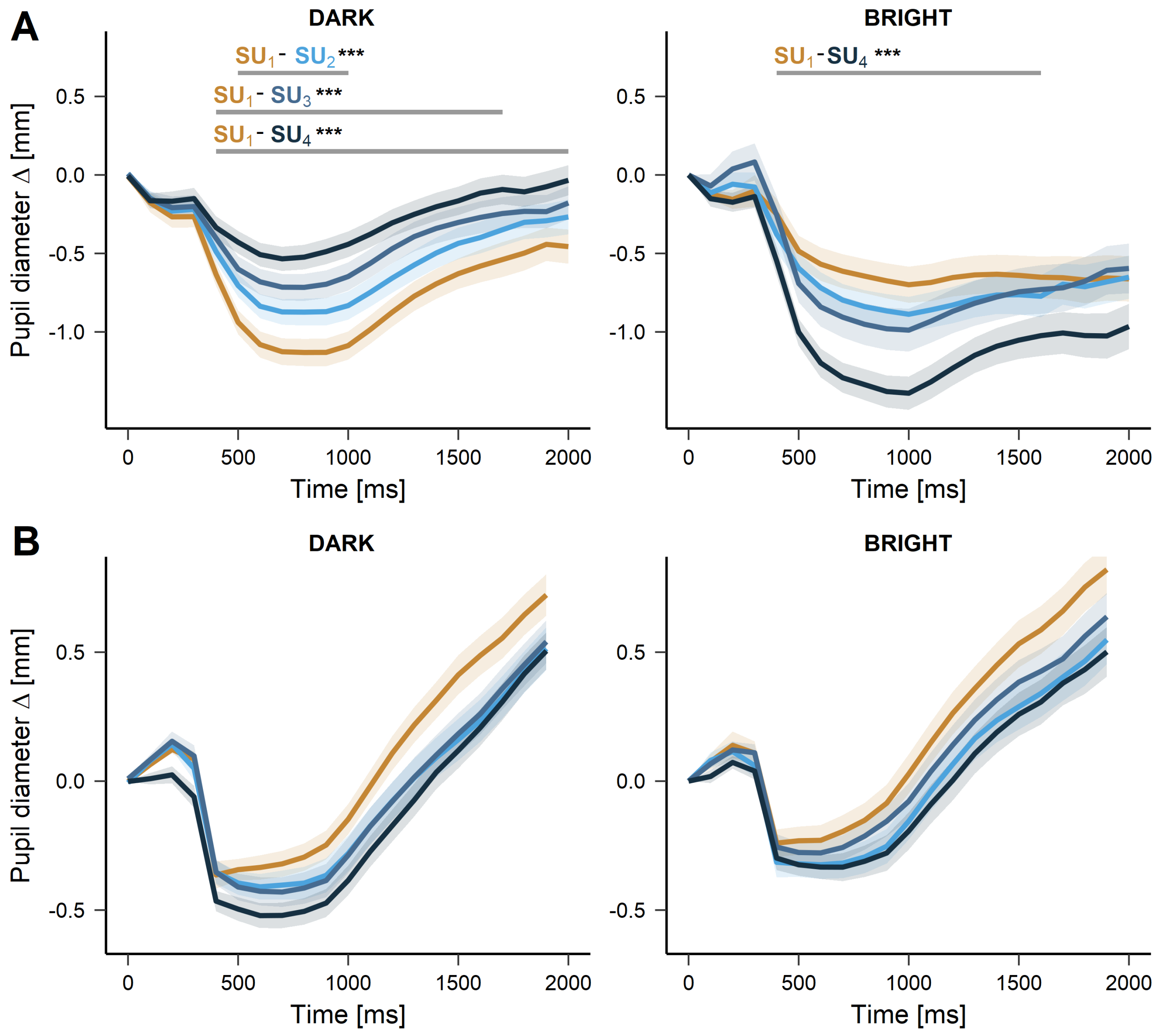

Figure S2. **A** Pupil size during the cue period as a function of time in *dark* (left panel) and *bright* (right panel) trials. Time zero on the X-axis shows cue onset time. Colored lines indicate baseline corrected mean pupil size change for different SUs. **B** Pupil size during the foreperiod as a function of time in *dark* (left panel) and *bright* (right panel) trials. Time zero on the X-axis represents the offset of the WS or the beginning of the foreperiod. Horizontal grey lines indicate clusters of differences between conditions (CBPT significance: $* \, p \leqslant .05$, $** \, p \leqslant .01$, $*** \, p \leqslant .001$).

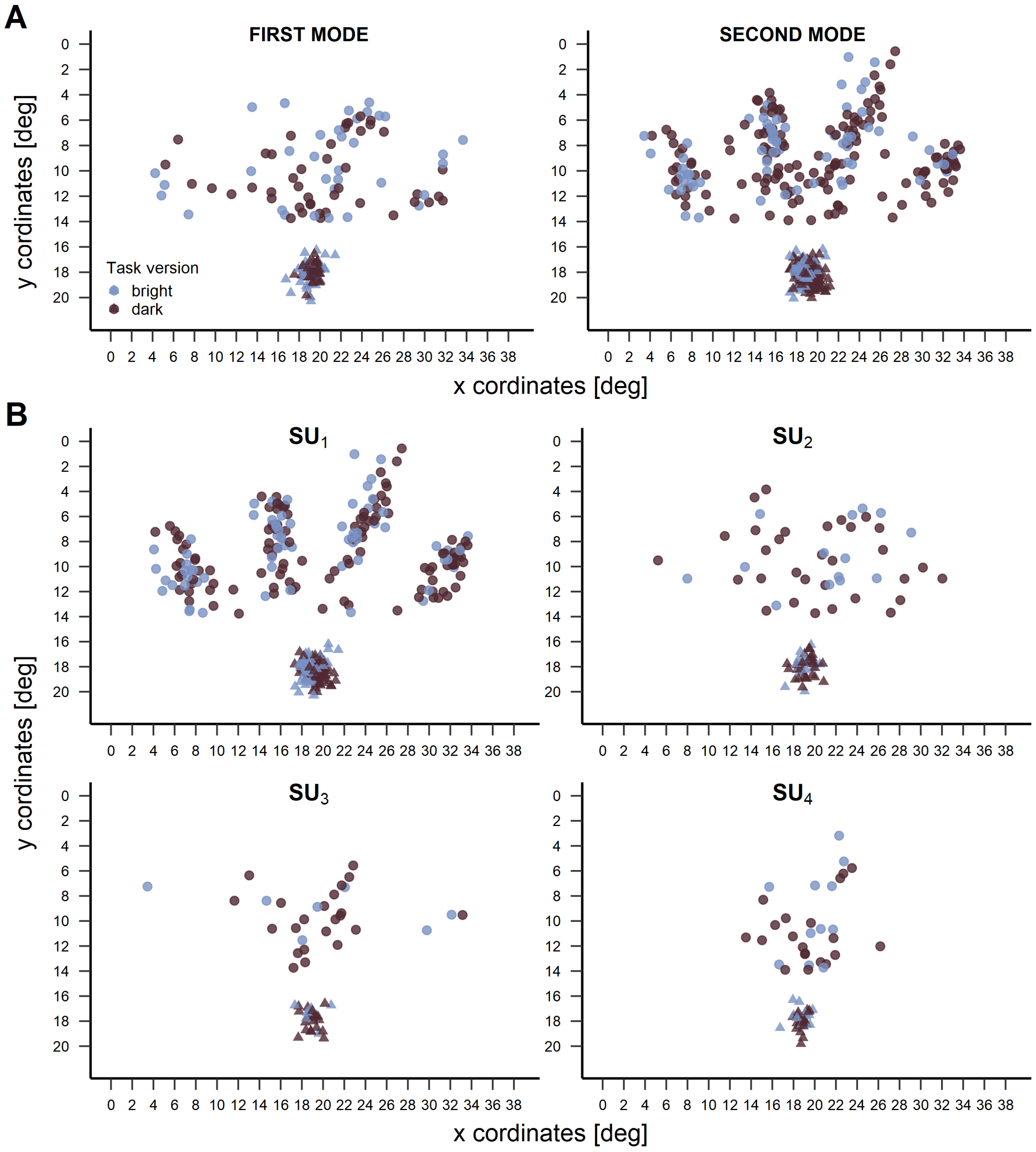

Figure S3. **A** Starting (triangles) and ending (dots) positions of saccades during the foreperiod. Different colors were used for the two versions of task (*bright* or *dark* cue). Saccades were grouped into first and second mode responses. **B** Anticipatory saccades grouped according to levels of expected surprise.

B. Amplitude and direction of 1st and 2nd mode foreperiod saccades according to cue spatial display.

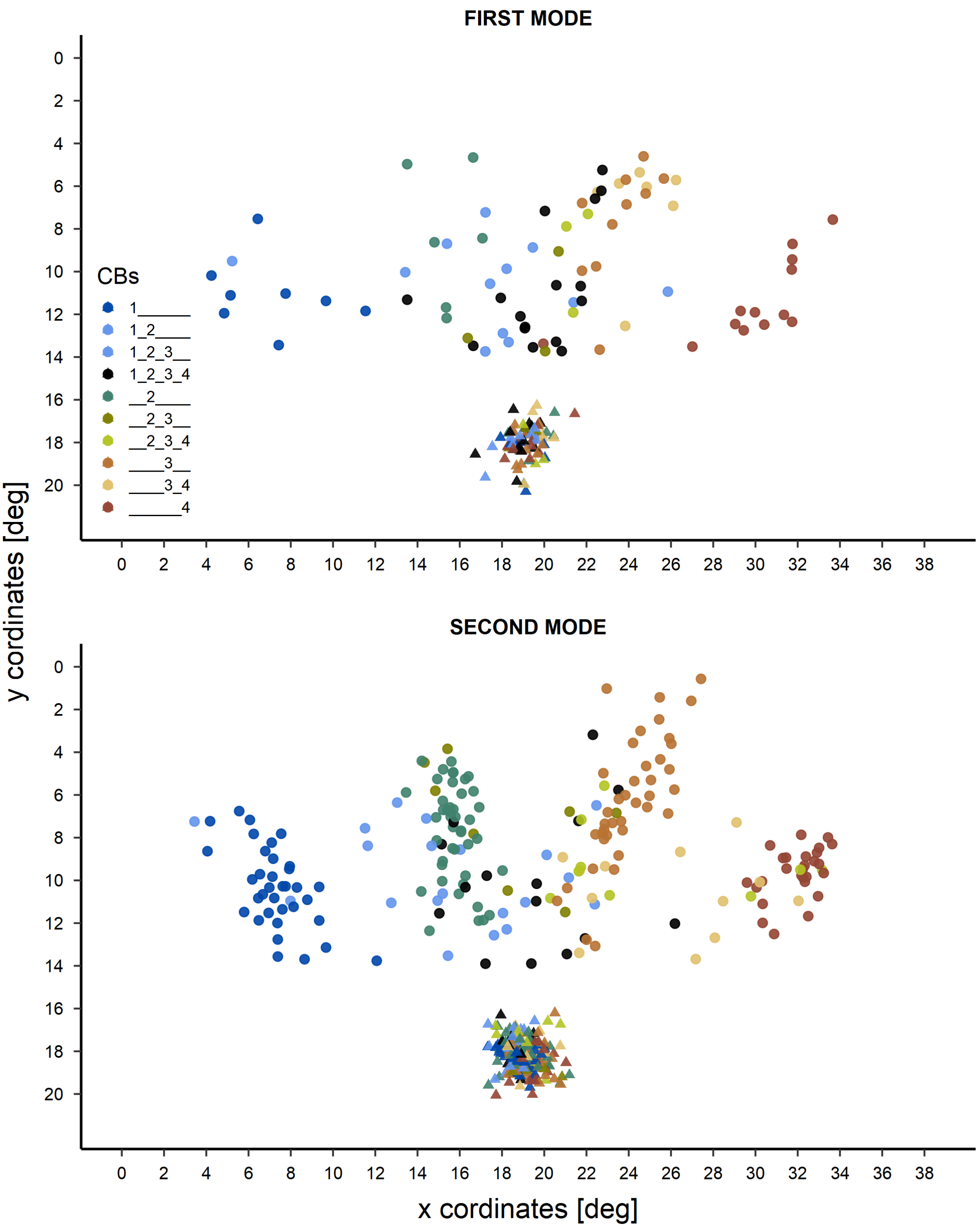

Figure S4. Starting (triangles) and ending (dots) positions of saccades during the foreperiod. Different colors used to indicate the spatial display of cues during the previous cue period. Numbers indicate CBs that were marked by the given cue. Saccades during the foreperiod were directed towards the area marked during the cue period.
